# Local application of osteoprotegerin-chitosan gel in critical-sized defects in a rabbit model

**DOI:** 10.7717/peerj.3513

**Published:** 2017-06-30

**Authors:** Soher N. Jayash, Najihah M. Hashim, Misni Misran, NA Baharuddin

**Affiliations:** 1 Department of Restorative Dentistry/Faculty of Dentistry, University of Malaya, Kuala Lumpur, Malaysia; 2 Department of Pharmacy/Faculty of Medicine, University of Malaya, Kuala Lumpur, Malaysia; 3 Centre For Natural Products And Drug Discovery (CENAR), Department of Chemistry, Faculty of Science, University of Malaya, Kuala Lumpur, Malaysia; 4 Department of Chemistry/Faculty of Science, University of Malaya, Kuala Lumpur, Malaysia

**Keywords:** Osteocalcin, Osteopontin, Cathepsin K, OPG-chitosan gel, Rabbit

## Abstract

**Background:**

Osteoprotegerin (OPG) is used for the systemic treatment of bone diseases, although it has many side effects. The aim of this study was to investigate a newly formulated OPG-chitosan gel for local application to repair bone defects. Recent studies have reported that immunodetection of osteopontin (OPN) and osteocalcin (OC) can be used to characterise osteogenesis and new bone formation.

**Methods:**

The osteogenic potential of the OPG-chitosan gel was evaluated in rabbits. Critical-sized defects were created in the calvarial bone, which were either left unfilled (control; group I), or filled with chitosan gel (group II) or OPG-chitosan gel (group III), with rabbits sacrificed at 6 and 12 weeks. Bone samples from the surgical area were decalcified and treated with routine histological and immunohistochemical protocols using OC, OPN, and cathepsin K (osteoclast marker) antibodies. The toxicity of the OPG-chitosan gel was evaluated by biochemical assays (liver and kidney function tests).

**Results:**

The mean bone growth in defects filled with the OPG-chitosan gel was significantly higher than those filled with the chitosan gel or the unfilled group (*p* < 0.05). At 6 and 12 weeks, the highest levels of OC and OPN markers were found in the OPG-chitosan gel group, followed by the chitosan gel group. The number of osteoclasts in the OPG-chitosan gel group was lower than the other groups. The results of the liver and kidney functional tests indicated no signs of harmful systemic effects of treatment. In conclusion, the OPG-chitosan gel has many characteristics that make it suitable for bone repair and regeneration, highlighting its potential benefits for tissue engineering applications.

## Introduction

The gold standard for bone regeneration is an autologous bone graft. However, the procurement of autogenous bone comes with some disadvantages, such as creating an additional surgical area, significant morbidity, and limited source material. Thus, bone tissue engineering and regenerative medicine can be employed to improve bone regeneration (Arrigoni et al., 2013; Muschler et al., 2010; Sasso et al., 1998). The discovery of osteoprotegerin (OPG) as an inhibitor of osteoclast activity and maturation has led to new research exploring the applicability of OPG as a potential therapeutic agent for the treatment of bone diseases and to induce bone formation (Bekker et al., 2001; Fili, Karalaki & Schaller, 2009; Hofbauer et al., 2001; Kostenuik et al., 2001). The first clinical trial evaluated the efficacy of recombinant Fc-OPG, used systemically as a drug for the treatment of osteoporosis in postmenopausal women (Bekker et al., 2001) and another study evaluated a different formulation of OPG, known as AMGN-0007, in patients with lytic bone lesions associated with multiple myeloma or breast carcinoma (Body et al., 2003). Both of these studies reported that Fc-OPG treatment resulted in reduced bone turnover markers when administered at a low dose, and had a longer antiresorptive effect when administered at an equivalent dose. The authors of these studies cited two potential concerns with Fc-OPG therapy. The first is the generation of anti-Fc-OPG antibodies, which might cross-react with endogenous Fc-OPG, neutralising its activity. The second potential concern is the binding of Fc-OPG to TNF-related apoptosis-inducing ligands, which could inhibit their role in tumour surveillance (Schwarz & Ritchlin, 2007). These side effects are, in part, due to systemic administration of the treatment. Recently, it was reported that twice-weekly injections with a high dose of OPG-Fc (5.0 mg/kg) into the mesial and distal mucosa of the first molars during orthodontic movement improved bone quantity and orthodontic anchorage in a rat model (Fernández-González et al., 2016). In addition, OPG-chitosan matrices have been shown to enhance cell growth and proliferation, as well as increasing the production of osteopontin (OPN) and osteocalcin (OC) protein levels (Jayash et al., 2016). In order to optimise the OPG concentration and prolong the duration of protein release, aimed at avoiding the side effects associated with systemic application, a controlled drug delivery system for the local application of OPG is required. Chitosan was selected as the matrix material for this method of drug delivery.

Bone matrix proteins such as OPN function to induce osteoclast migration and adhesion, while OC functions to regulate mineralisation. These functions highlight the importance of measuring bone matrix proteins when characterising osteogenesis processes, as well as the influence of the drug on their expression (Bondarenko et al., 2014). Cathepsin K is one of the biomarkers expressed by osteoclasts during active bone resorption, making it a useful and specific biomarker of osteoclastic activity. Cathepsin K is expressed by osteoclasts and a small number of osteoclast precursors, but it is not expressed by osteoblasts or osteocytes. Therefore, its expression is specific to the resorption phase of bone metabolism (Drake et al., 1996).

We previously formulated and investigated an OPG-chitosan gel for its cytocompatibility (Jayash et al., 2017a); however, the *in vivo* effects on bone regeneration were not extensively investigated and discussed in our previous work. In the present paper, we investigated the efficacy of an OPG-chitosan gel in bone regeneration in terms of the expression of osteoblast- and osteoclast-specific proteins, and tested the toxicity of the OPG-chitosan gel by biochemical assays (liver and kidney function tests).

## Methods

### Formulation of the osteoprotegerin-chitosan gel

The OPG-chitosan and chitosan gels were prepared using water-soluble chitosan (25 kDa). Recombinant human OPG protein (1 mg/mL) was used to prepare the OPG-chitosan gel (PeproTech, Rocky Hill, NJ, USA) with a chitosan binder (85 kDa). The methodology for preparing the OPG-chitosan gel has been described previously (Jayash et al., 2017b) and patented under the title, “An osteoprotegerin-chitosan gel for bone tissue regeneration” (PI 2016701598,UM).

### Animal model

The animal experiment was authorised by the Institutional Animal Care and Use Committee at the University of Malaya (FOM IACUC), and registered under number 20150115/DENT/R/NAB. Critical-sized defects were created in the calvarial bone of 18 New Zealand white female rabbits (6 months old; 3.5–4 kg). The rabbits were divided into three groups: (i) untreated control group (group I; *n* = 6), (ii) chitosan-only gel (group II; *n* = 6), and (iii) OPG–chitosan gel (group III; *n* = 6). The anaesthesia, surgery, medical treatment, and euthanasia procedures have been described previously (Jayash et al., 2017a). Three rabbits from each group were examined at 6 and 12 weeks. The bone samples were fixed in 10% neutral buffered formalin for 24 h, decalcified in 10% EDTA for 3 weeks, then embedded in paraffin according to the established technique. Three 4-µm thick sections from each paraffin block were cut using a microtome (Leica, Wetzlar, Germany). For histological studies, the sections were stained with haematoxylin and eosin (H&E). Immunohistochemistry was performed according to established methods (Gruber & Ingram, 2003; Bondarenko et al., 2014). The anti-OPN (clone 1B20; Novus Biologicals, Cambridge, UK), anti-OC (OCG3; Abcam, Cambridge, UK), and cathepsin K (Biovision, Milpitas, CA, USA) polyclonal antibodies have previously been verified in rabbit bone tissue (Arrigoni et al., 2013; Bondarenko et al., 2014).

### Quality control

A negative control reagent was used with each specimen to identify any non-specific staining. If non-specific staining could not be clearly differentiated from specific staining, the labelling of the test specimen was considered invalid. In this experiment, rabbit immunoglobulin fraction (normal; Dako, Industrial RowTroy, Michigan, USA) was used as the negative control.

### Histological evaluation

Verification of the immunohistochemical reaction was performed using a light microscope and scanned using a digital slide scanner (3DHISTECH Ltd., Budapest, Hungary). The results were assessed using computer-assisted image analysis (ImageJ; National Institutes of Health, Bethesda, MD, USA). The image was opened and the image threshold was adjusted until all stained areas were selected. A histogram was displayed to provide assistance. Staining was assessed by setting a threshold using the threshold tool. The threshold tool settings that successfully quantified the staining in a positive-stained specimen were repeated in every image for comparison. The analysed–set measurement was selected, and the parameters to be measured were chosen (Jensen, 2013). The mean and standard deviation were calculated for each sample.

### Serum biochemical parameters

Blood samples were collected from all rabbits before surgery and before sacrifice. The blood samples were allowed to clot at room temperature before centrifuging at 1,000 g for 10 min. The serum was separated and analysed for markers of kidney function, including creatinine and urea nitrogen, and markers of liver function, including alkaline phosphatase (ALP) and alanine aminotransferase (ALT). Analyses were conducted using a clinical chemistry analyser (902; Hitachi, Tokyo, Japan) with standard diagnostic kits (Hitachi 902, Roche).

### Statistical analysis

Statistical analyses were performed using the parametric one-way ANOVA test and non-parametric Mann–Whitney *U* and Kruskal–Wallis tests for comparison between the mean values of different groups. The significance value was set at *p* < 0.05. Results are presented as the mean (arithmetic mean) and standard deviation.

## Results

### Clinical findings

**Figure 1:**
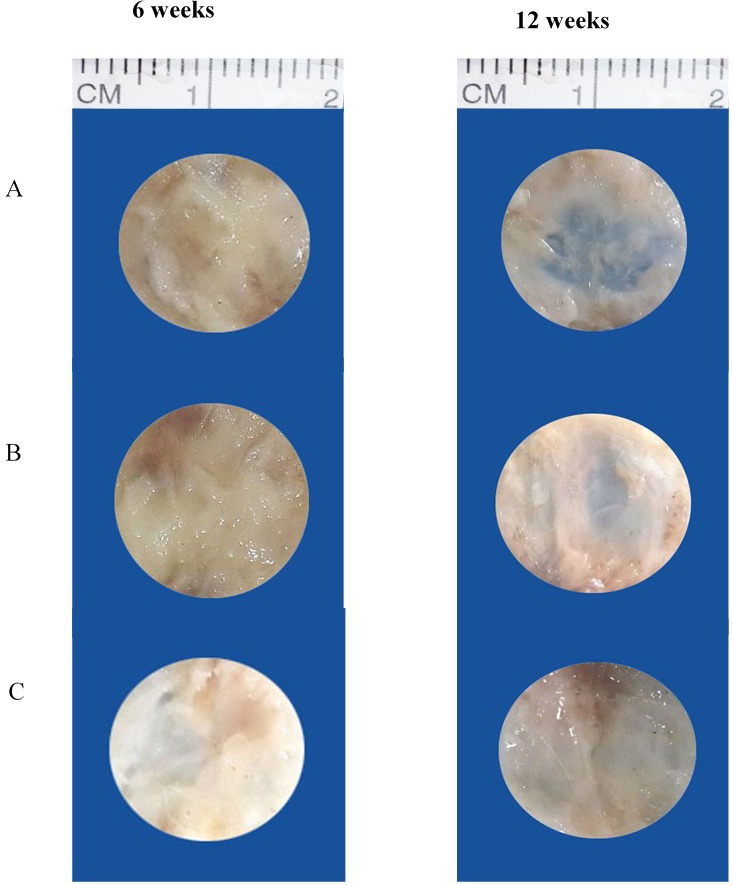
Gross appearance of surgical defects at 6 and 12 weeks. (A) Group I (control), (B) group II (chitosan gel), and (C) group III (OPG-chitosan gel).

At 6 weeks, rabbits in group III (OPG-chitosan gel) showed partially healed surgical defects, which were filled with a dense, opaque structure (bone). On the other hand, the surgical defects of groups I (surgery only) and II (chitosan gel) were completely filled with thin, transparent soft tissue. At 12 weeks, the surgical defects of groups II and III were completely filled with hard tissue, whereas the defects of group I were still only partially healed, with regions of soft tissue (Fig. 1).

### Histological results (haematoxylin and eosin staining)

At 6 weeks, the defects of rabbits in group III were filled with new bone and osteoid tissue, while the group II defects were filled with new bone and fatty marrow. However, the group I defect was only partially filled, with the least amount of new bone compared to groups II and III. The connective tissue of the bone bridge in groups II and III was less prominent than that of group I. No graft particles were seen at 6 weeks, which suggests that the particles may have been completely resorbed. The new osteoid bone filling each of the defects was formed within the region of interest. The trabecular bone in group II appeared thick and dense, while the trabecular bone in group III had become lamellar bone.

**Figure 2:**
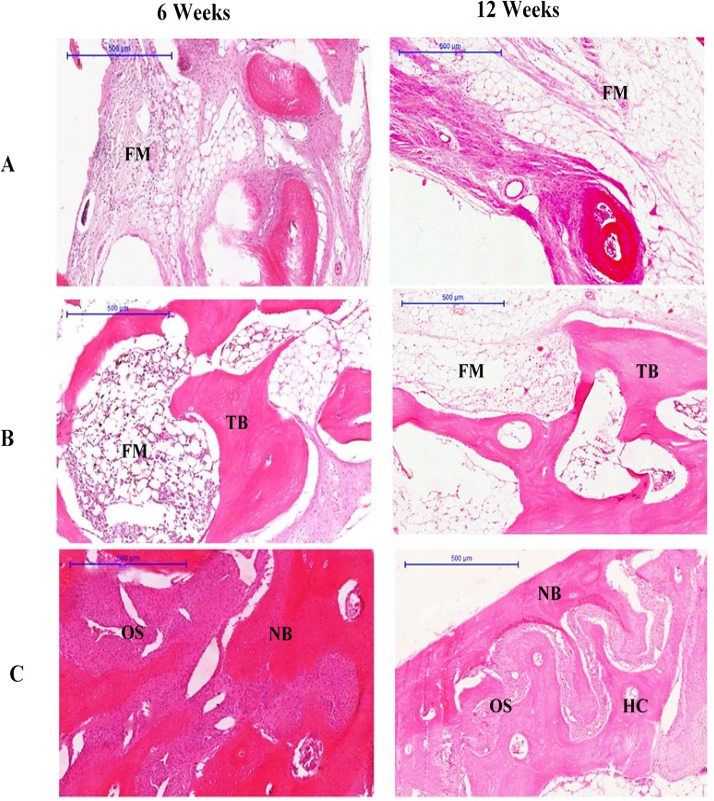
Photomicrographs of defect sites of (A) Group I, (B) Group II (C) Group III at 6 and 12 weeks. Sections were stained with haematoxylin and eosin. NB, new bone; OS, osteoid; FM, fatty marrow; HC, Haversian canal; TB, trabecular bone. (Scale bar represents 500 µm.)

At 12 weeks, newly formed bone had completely filled the defect of rabbits in group III. The newly formed bone in these defects resembled a bridge, and was arranged as lamellae in some areas. These areas contained large osteons and Haversian canals, as well as highly cellularised connective tissue, especially within the central region. Moreover, cortical organisation was evidenced by the presence of trabecular projections, in addition to the maturation of lamellar bone indicated by thickening at the margin of the defect compatible with the original bone structure of the region (Fig. 2).

### Immunohistochemistry results

#### Osteoblast markers

**Figure 3:**
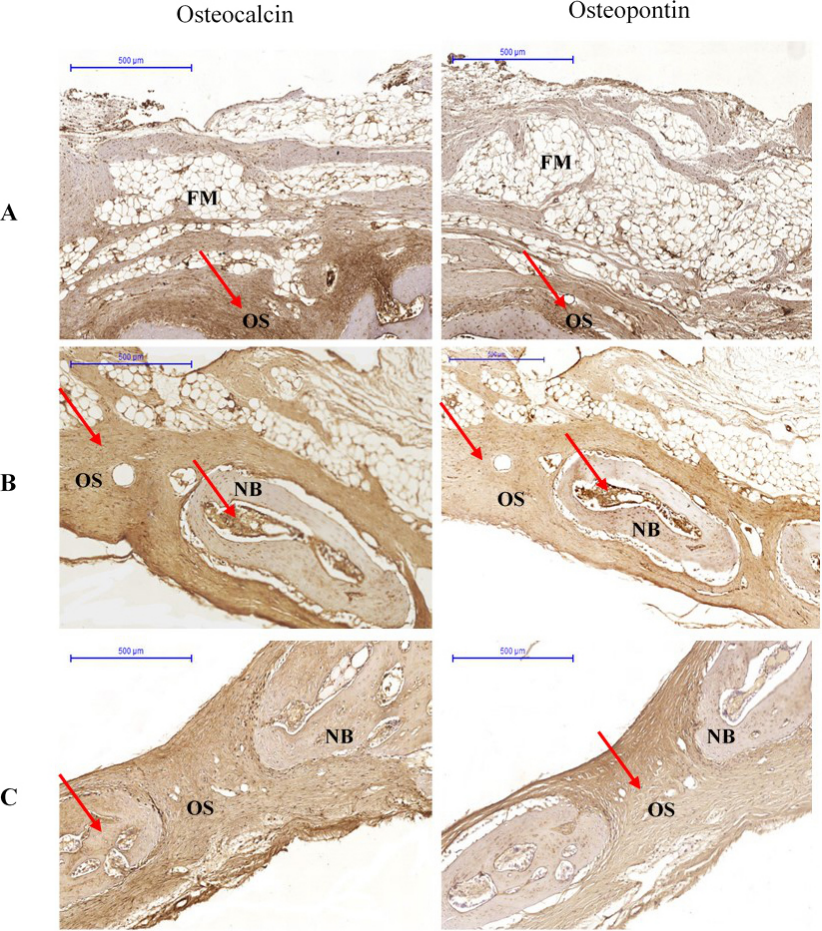
Photomicrographs of immunostaining for osteocalcin and osteopontin in (A) Group I, (B) Group II (C) Group III at 6 weeks. The pictures are arranged by staining technique (columns) and the investigated treatment (rows). Areas that stained positive for osteocalcin and osteopontin are indicated by red arrowheads. NB, new bone; OS, osteoid; FM, fatty marrow.

At 6 weeks, OPN immunolabelling was observed in the osteoclasts, osteoblasts, and fibroblasts. In almost all specimens, OPN immunoreactivity was located within the matrix of compact bone, cancellous bone, and osteoid. Osteocalcin immunoreactivity appeared in the matrix of compact bone, but was weaker in cancellous bone and osteoid (Fig. 3).

**Figure 4:**
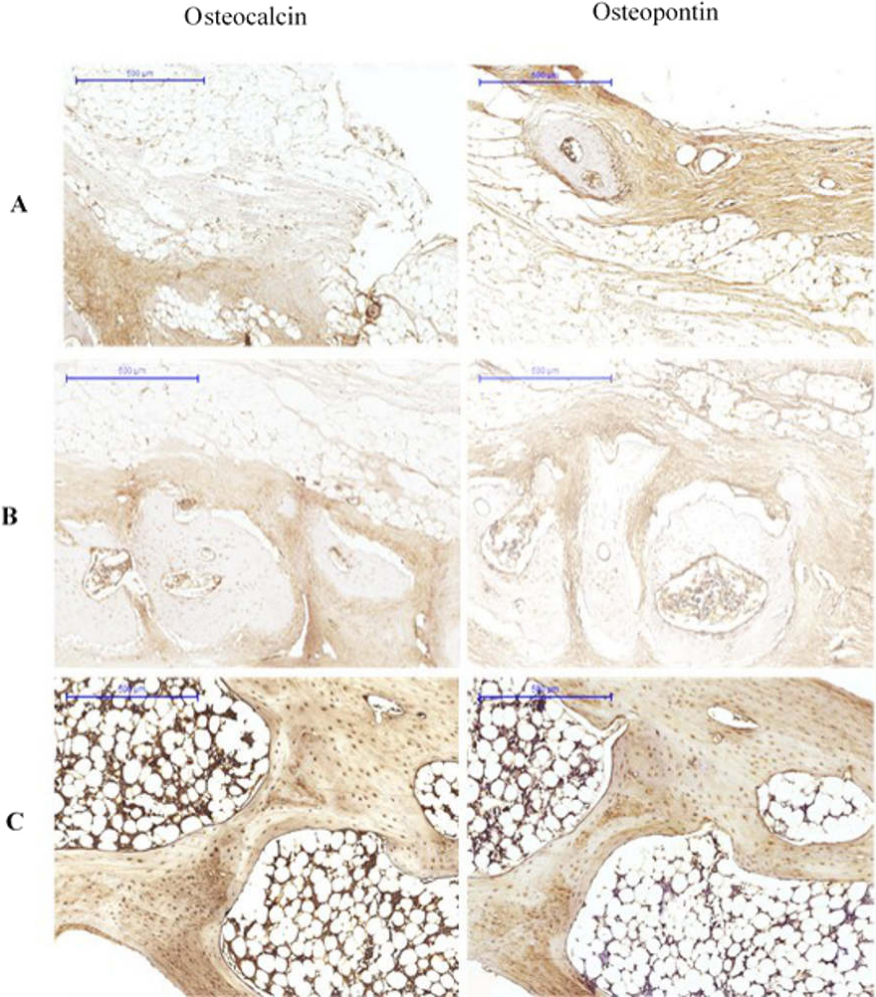
Immunohistological results at 12 weeks for (A) Group I, (B) Group II (C) Group III. The pictures are arranged by staining technique (columns) and by the investigated treatment (rows). Areas that stained positive for osteocalcin and osteopontin are indicated by red arrowheads. NB, new bone; OS, osteoid; FM, fatty marrow; CB, compact bone; CAB, cancellous bone.

At 12 weeks, OPN expression was characterised by osteoid staining in all investigated groups. Immunolabelling of OPN was observed in osteoblasts. The bone matrix also showed stained areas. Osteocalcin was detected in the matrix of compact bone, but showed reduced expression in osteoid and bone cells (Fig. 4).

**Figure 5:**
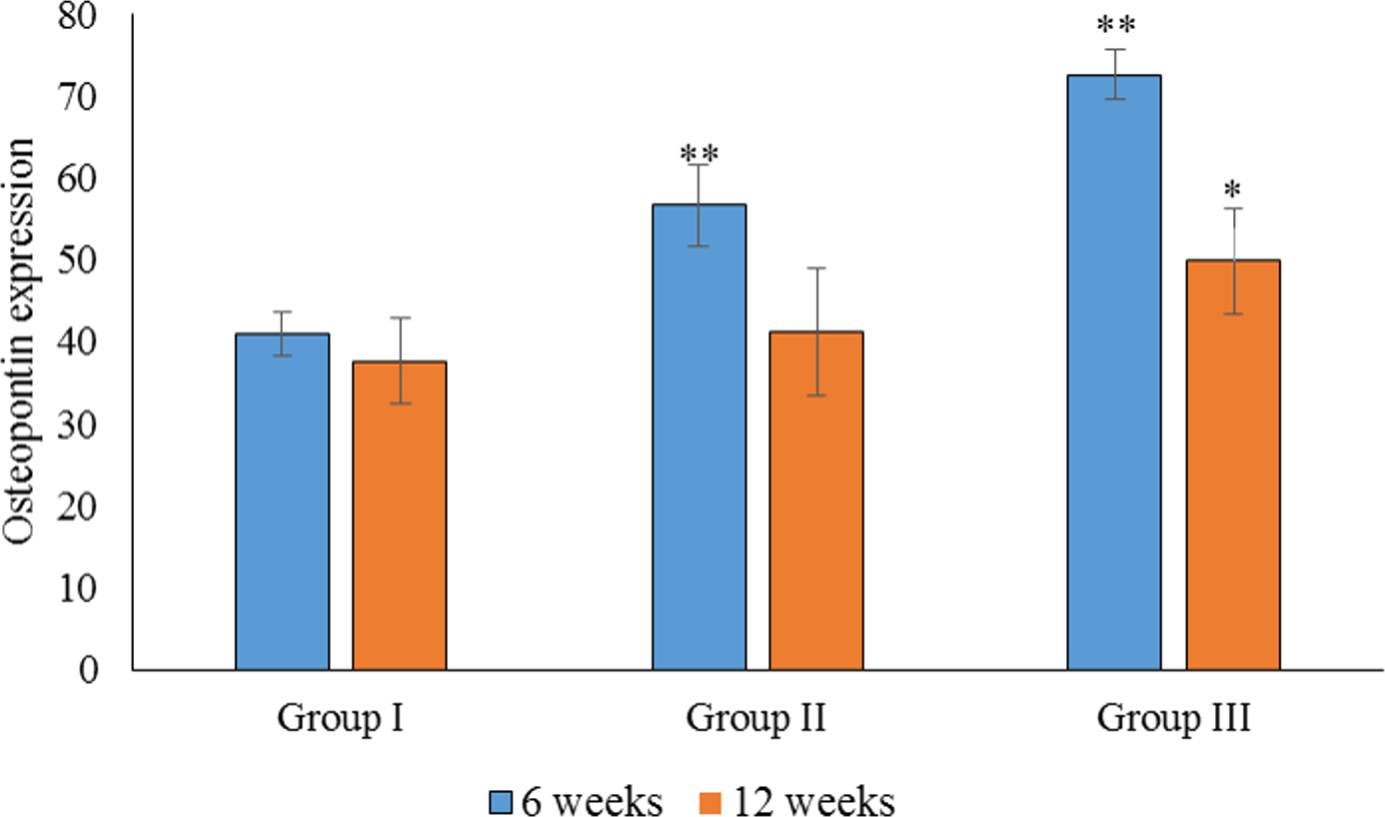
Statistical analysis of the percentage expression of osteopontin as a bone-formation marker between groups I, II and III at 6 and 12 weeks. Data are presented as the average of three independent experiments (*n* = 3). ^∗∗^Significant difference between the control (group I) and experimental groups (groups II and III). ^∗^Significant difference between groups III and II. A *p*-value of <0.05 was considered significant in all analyses.

Based on the parametric one-way ANOVA, there was a significant difference in the mean percentage of OPN expression between groups I and III (*p* < 0.05). The highest expression of OPN was observed in group III, followed by group II and group I, which showed the lowest OPN expression. Based on the parametric one-way ANOVA, there was no significant difference in the mean percentage of OPN expression between groups II and I (*p* > 0.05). However, there was a significant difference in the mean percentage of OPN expression between groups III and II (*p* < 0.05; Fig. 5).

**Figure 6:**
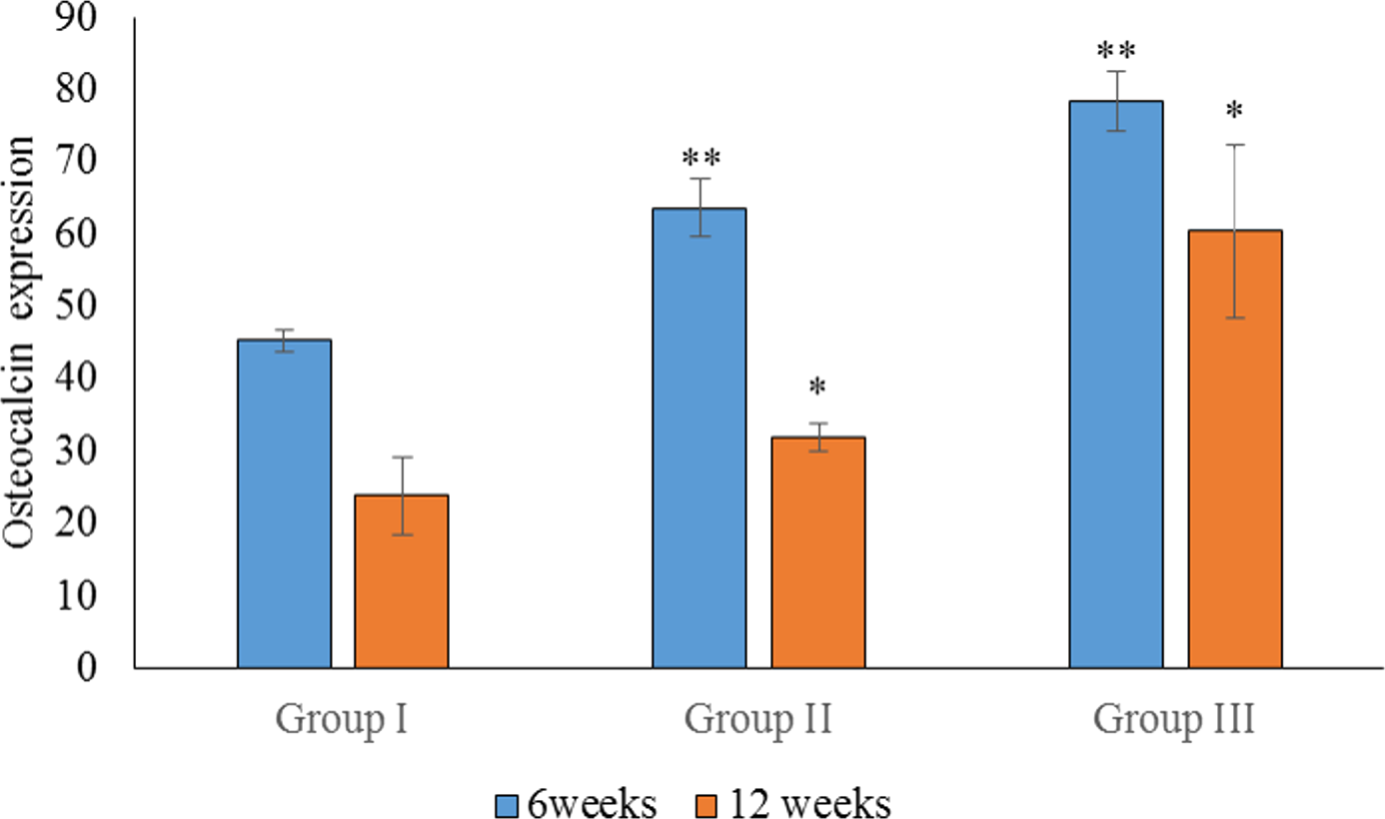
Statistical analysis of the percentage expression of osteocalcin as a bone-formation marker between groups I, II and III at 6 and 12 weeks. Data are presented as the average of three independent experiments (*n* = 3). ^∗∗^Significant differences (*p* < 0.05) between control (group I) and experimental groups (groups II and III).

Statistical analysis of the percentage of OC expression at 6 weeks showed a significant difference between groups I, II and III (*p* < 0.05). The highest OC expression was found in group III, followed by group II (Fig. 5). Regarding OC expression at 12 weeks, there was a significant difference in the mean percentage of OC expression in group III when compared to groups II and I (*p* < 0.05). There was also a significant difference in the mean percentage of OC expression between groups III and I (*p* < 0.05). The highest OC expression at 12 weeks was found in group III, followed by group II (Fig. 6).

In the intragroup comparison, there was a significant difference in OPN expression between 6 and 12 weeks in all groups (*p* < 0.05), with the highest expression of OPN observed at 6 weeks. Similarly, there was a significant difference in OC expression between 6 and 12 weeks in all groups, with the highest expression observed at 6 weeks.

The expression of OPN and OC between 6 and 12 weeks was compared for all groups. We found no significant difference in the expression of OPN and OC in group III (*p* > 0.05). In group II, however, there was a significant difference in the expression of OPG and OC at 6 weeks (*p* < 0.05), when OC was more highly expressed. In group I, there was a significant difference between the expression of OPN and OC at 12 weeks, with higher expression observed for OPN. As the sample size was small, non-parametric tests were also carried out, and similar results were obtained.

#### Osteoclast marker

**Figure 7:**
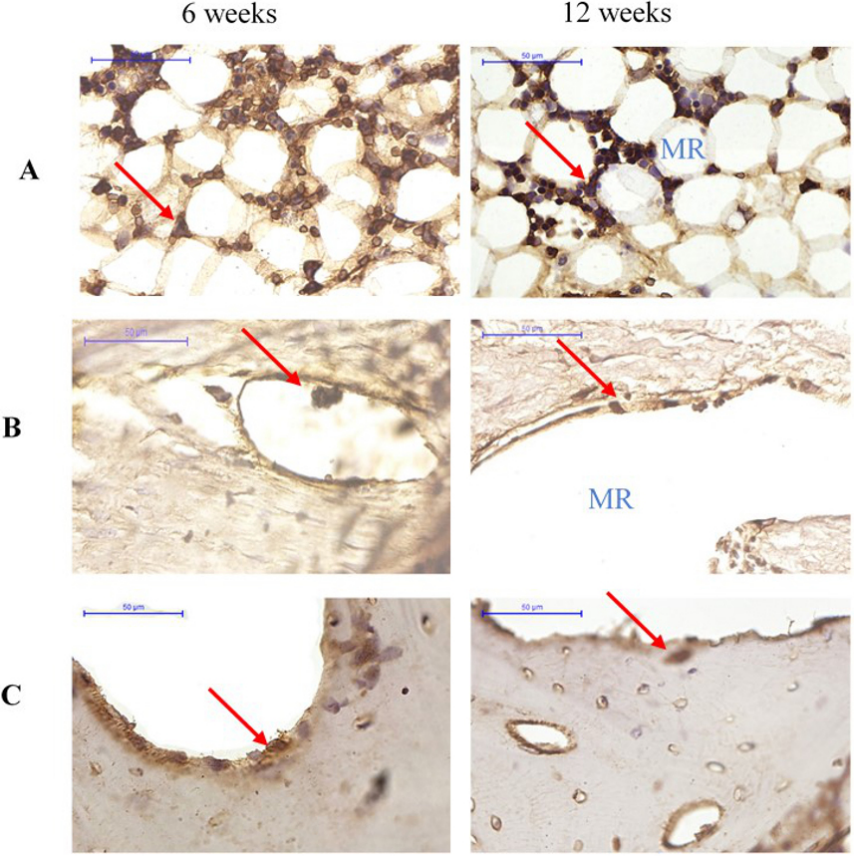
Cathepsin K immunostaining of osteoclasts in groups (A) Group I, (B) Group II (C) Group III at 6 and 12 weeks after surgery. Cathepsin K-positive multinuclear cells are indicated by red arrowheads. MR, medullary region.

More cathepsin K-positive areas were observed in the medullary region of the defect site than the cortical bone region at both 6 and 12 weeks. Cathepsin K-positive multinuclear cells were also detected within the newly-formed bone. In addition, fewer osteoclasts were detected at 12 weeks than at 6 weeks for all groups (Fig. 7).

#### Serum biochemical parameters

The serum biochemistry results of rabbits in groups I, II and III at baseline and at 6 and 12 weeks are summarised in Tables 1 and 2. There were no significant changes in the levels of creatinine and urea nitrogen. In addition, no significant differences in serum electrolytes including calcium, potassium, and chloride were noted. The effects of OPG and/or chitosan on liver function parameters such as albumin, ALT, G-glutamyl transferase, ALP and total bilirubin were also examined. Animals in groups I, II and III showed no difference in hepatic markers. The effects of treatment on triglyceride, high-density lipoprotein (HDL) cholesterol, and total cholesterol levels are shown in Table 2. Rabbits in all groups showed no significant changes in triglyceride, HDL cholesterol, or total cholesterol levels after treatment.

## Discussion

Allografts and autografts present several clinical problems that need to be addressed, such as transmission of infection, unpredictable resorption, pain at the donor site, and limited donor source. To eliminate these problems, it would be advantageous to develop a material that can promote bone regeneration at the defect site. The intention of this study was to investigate the local of application of OPG-chitosan gel in a critical-sized defect in the parietal bone in a rabbit models. To investigate this, three treatments (OPG-chitosan gel, chitosan gel, and untreated control groups) were tested at 6 and 12 weeks. The clinical results of the current study showed that treatment of surgical defects with the OPG-chitosan gel was not associated with any signs of inflammation or infection. This indicates biocompatibility of the newly formulated OPG-chitosan gel. At 12 weeks, the surgical defects of rabbits treated with the chitosan gel were filled with new bone, as observed from the histological sections. However, the defects in this group were only partially filled, indicating that chitosan enhances bone formation. This observation is in agreement with previous studies that reported a higher amount of bone formed in defects treated with chitosan compared to untreated controls (Ezoddini-Ardakani et al., 2012). In addition, the amount of bone formed was reduced when compared to those defects treated with chitosan combined with other active components (Bush et al., 2016; Oktay et al., 2010).

**Table 1:** Serum biochemical data for rabbits treated with OPG-chitosan (group III) or chitosan (group II) gels and untreated control rabbits (group I) at baseline and 6 weeks after treatment.

Parameter	Baseline			6 weeks			Normal range
	Group III	Group II	Group I	Group III	Group II	Group I	
Sodium mmol/L	142 ± 0	142 ± 1	141 ± 1	143 ± 0	141 ± 1	141 ± 0.7	139–146
Potassium mmol/L	4 ± 0.1	5 ± 0.4	5 ± 0.7	3.8 ± 0.1	3.95 ± 0.1	5 ± 0.6	3–5
Chloride mmol/L	103 ± 0.7	102 ± 0	101.5 ± 0.2	100 ± 4.2	101 ± 1	99.5 ± 4	104–116
Urea nitrogen mmol/L	8 ± 0.42	7 ± 1.5	8 ± 0.2	6 ± 1.5	7 ± 0.07	9 ± 0.07	6.35–16
Creatinine µmol/L	77 ± 6	78 ± 2	77 ± 5	102 ± 15	77 ± 7	85 ± 4	60–140
Albumin (g/L)	41 ± 0.0	38 ± 1	41 ± 1	39 ± 4	40 ± 4	40 ± 1	20–40
Total Bilirubin (µmol/L)	<2	<2	<2	<2	<2	<2	2–5
Alkaline Phosphate (U/L)	102 ± 6	111 ± 8	110 ± 4.9	50 ± 10	81 ± 3	69 ± 3	17–192
Alanine Aminotransferase (U/L)	30.5 ± 3	94.5 ± 2	36 ± 15	35 ± 0	53 ± 12.7	52.5 ± 3.1	38–86
G-Glutamyl Transferase (U/L)	5.5 ± 0.7	5.5 ± 0.9	2.5 ± 0.7	6 ± 1.5	6.5 ± 1.4	5 ± 1.4	6–22
Triglyceride (mmol/L)	0.5 ± 0.1	0.7 ± 0.1	0.65 ± 0.2	0.5 ± 0.1	0.7 ± 0.15	0.75 ± 0	–
Total Cholesterol (mmol/L)	1 ± 0.2	0.9 ± 0.1	1.1 ± 0	1.3 ± 0.3	0.85 ± 0.2	1.2 ± 0.1	–
HDL Cholesterol (mmol/L)	0.8 ± 0.2	0.61 ± 0.1	0.8 ± 0.2	0.2 ± 0.1	0.62 ± 0.1	0.8 ± 0.1	–

**Notes.**

Data are expressed as the mean ± SD of three rabbits per group.

**Table 2:** Serum biochemical data for rabbits treated with OPG-chitosan (group III) or chitosan (group II) gels and untreated control rabbits (group I) at baseline and 12 weeks after treatment.

Parameter	Baseline			12 weeks			Normal range
	Group III	Group II	Group I	Group III	Group II	Group I	
Sodium mmol/L	142 ± 2	142 ± 1	143 ±2	142 ± 2.6	143 ± 1	143 ± 2	139.3–145.7
Potassium mmol/L	4 ± 1	4 ± 0.3	4 ± 0.4	4 ± 0.6	4 ± 0.4	4 ± 0.5	3–5
Chloride mmol/L	100 ± 0.5	101 ± 0.6	98 ± 3.5	102 ± 0.2	101 ± 2	104 ± 4	105–116
Urea nitrogen mmol/L	8 ± 1	8 ± 1	9 ± 0.4	8 ± 1	7 ± 1	8 ± 1	6–16
Creatinine µmol/L	85.7 ± 10	87 ± 1	89 ± 2	89 ± 10	83 ± 2	87 ± 1	60–140
Albumin (g/L)	43.3 ± 3	40.7 ± 1	42 ± 1	41 ± 2.9	40 ± 3	39 ± 3	20–41
Total Bilirubin (µmol/L)	<2	<2	<2	<2	<2	<2	2–5
Alkaline Phosphate (U/L)	68.5 ± 12	44 ± 3	69.5 ± 8	58 ± 1	44 ± 9	48 ± 8	17–192
Alanine Aminotransferase (U/L)	58 ± 6.9	60 ± 9.7	48.7 ± 10	53.7 ± 15.8	67 ± 12	54 ± 7	38–86
G-Glutamyl Transferase (U/L)	6 ± 1.2	7 ± 1.5	3 ± 1.4	4 ± 0.6	7 ± 1.7	5 ± 1.2	6.00–22.00
Triglyceride (mmol/L)	0.7 ± 0.5	0.6 ± 0.2	0.4 ± 0.01	1.2 ± 0.2	0.6 ± 0.2	0.5 ± 0.2	–
Total Cholesterol (mmol/L)	1 ± 0.2	1 ± 0.2	1 ± 0.2	1 ± 0.2	1 ± 0.2	1 ± 0.2	–
HDL Cholesterol (mmol/L)	0.7 ± 0.1	0.5 ± 0.03	0.7 ± 0.1	0.9 ± 0.04	0.6 ± 0.1	0.6 ± 0.1	–
LDL Cholesterol (mmol/L)	0.2 ± 0.1	0.2 ± 0.08	0.1 ± 0.1	0.1 ± 0.1	0.2 ± 0.1	0.26 ± 0.1	–

**Notes.**

Data are expressed as the mean ± SD of three rabbits per group.

Our observation that the surgical defects treated with OPG-chitosan gel (group III) were completely filled with new bone suggests that the OPG protein represents an active component that could improve bone regeneration. We also observed a large number of osteons in the bone tissue of the OPG-chitosan group at 6 and 12 weeks. This observation is in agreement with an earlier study (Allegrini et al., 2006), and supports the notion that active bone formation is taking place during the healing process. In any bony defect, the most intensive cellular reactions occur during the first 6 weeks, when the bone defect is initially healed with trabecular bone consisting of primitive woven bone. After 6 weeks, there is a reduction in the number of cells in the area of the defect, as well as increased calcium deposition, which is in agreement with a study by Gehrke (2013). Bone remodelling was also active after 8 weeks of healing in the rabbit, with various degrees of bone maturation in addition to uneven osseous formation (Jansen et al., 1991).

The compatibility and osteoconductivity potential of the OPG-chitosan gel was evident from the presence of progressive new bone formation and remodelling in the surgical defects at 6 and 12 weeks. A previous study reported an osteoinductive effect of treatments used to enhance bone formation in rabbits through the increased expression of bone-formation proteins (e.g., osteopontin and collagen type I) in bone defects treated with the bioconstruct (Arrigoni et al., 2013).

The current study showed significantly stronger expression of OPN in the OPG-chitosan gel group compared to the control group, which supports the osteogenic potential of the OPG-chitosan gel. All groups showed higher expression of OPN at 6 weeks compared to 12 weeks, but no significant difference was observed for OC expression in the OPG-chitosan gel group. This is in agreement with other studies that found that OPN is expressed throughout matrix maturation, and OC seems to play a role in the early stages of bone formation, showing positive expression at the area of bone formation at 6 weeks (Al-Ghani, Al-Hijazi & AL-Zubaydi, 2011; Kim et al., 2015).

In this study, OC expression was associated with mature bone, confirming its expression as a late marker of osteogenesis. Osteocalcin is commonly localised adjacent to osteoblasts, osteoid, and within osteocyte lacunae. Tera et al. (2014) observed OC staining in bone defects in a rat model, and concluded that the staining was relatively weaker in the regenerating bone compared to mature bone. The expression of OC in OPG-chitosan, chitosan, and control groups was higher at 6 weeks compared to 12 weeks, except for OC expression in the OPG-chitosan gel group, for which there was no significant difference. Osteocalcin appears to play a role in the early stages of bone formation, and showed positive expression at the area of bone formation at 6 weeks. The OC expression level was directly related to the bone formation activity.

The results of this study confirm the important roles of OC and OPN in bone healing, consistent with a study by Bondarenko et al. (2014). Evaluation of the biocompatibility and osteoconductivity of the OPG-chitosan gel demonstrated the superiority of this gel in terms of biocompatibility (no adverse reactions were observed), osteoconductivity, and progressive osteogenesis during the entire bone-healing period. Complete wound closure of the critical-sized bone defect filled with OPG-chitosan gel compared to chitosan gel and the unfilled control further supports the favourable biological properties of this biomaterial in a clinical situation. The immunohistological investigations presented here are consistent with enhanced new bone formation in the OPG-chitosan group, as we observed increased expression of OPN and OC when compared to the chitosan gel group and the untreated control group. This can be explained by the characteristics of the OPG-chitosan gel, which allows prolonged retention of OPG at the defect site. We demonstrated that OPG is released from the OPG-chitosan gel *in vivo*, and it is possible that the controlled release of OPG at the defect site enhances the recruitment of active osteoblasts and suppresses the recruitment of osteoclasts, thereby improving bone regeneration. We previously reported that the OPG-chitosan gel increases the *in vitro* proliferation of normal human osteoblasts (Jayash et al., 2017a).

Cathepsin K is expressed by osteoclasts and osteoclast precursors, but is absent in osteoblasts and osteocytes. Therefore, its expression is specific to the resorption phase of bone metabolism (Drake et al., 1996). Osteoblasts control the formation and activity of osteoclasts, as well as the resorption of bone through coupling mechanisms such as RANK, RANKL, and OPG (Jeon et al., 2016; Pederson et al., 2008; Teti, 2013).

The cathepsin K immunohistochemical staining showed fewer osteoclasts in the defect treated with OPG-chitosan gel than in the chitosan gel and control groups. This may be due to the action of OPG, which acts as a decoy receptor for RANKL, blocking the activation and maturation of osteoclasts. This observation is in agreement with previous studies in which chitosan has been demonstrated to enhance bone growth in critical-size defects in rat, rabbit, and sheep models (Muzzarelli et al., 1994; Pang et al., 2005; Wang et al., 2002). Previous studies have also shown a significant increase in bone formation in the osseous healing area of rats, rabbits, dogs, and humans where the OPG or chitosan-based biomaterial was applied (Ezoddini-Ardakani, 2011; Florczyk et al., 2013; Jung et al., 2014; Lamoureux et al., 2007; Yao et al., 2011). Furthermore, the serum levels of markers of liver and kidney function indicated no signs of any harmful systemic effects of treatment. This is in agreement with a previous study that reported no adverse effects in the liver and kidney resulting from administration of a chitosan drug for treating bone fractures (Ho et al., 2015).

## Conclusion

We conclude that the bioresorbable OPG-chitosan material induced the formation of a significant quantity of bone in a critical-sized parietal bone defect in a rabbit model. Although the chitosan gel group also yielded a greater amount of bone compared to the control group at all time points, the amount of bone generated was significantly lower than the OPG-chitosan gel at both 6 and 12 weeks. This study also reports that the OPG-chitosan gel showed the best behaviour, both clinically and histologically. It is biocompatible when used *in vivo*, and showed no adverse reactions. The combination of OPG and chitosan in a gel resulted in significantly enhanced new bone formation compared to chitosan alone or the untreated control in a rabbit model. This suggests that the enhanced new bone formation can be attributed to local release of OPG. The OPG-chitosan gel has many characteristics that make it suitable for bone repair and regeneration, highlighting its potential benefits for tissue engineering applications.

##  Supplemental Information

10.7717/peerj.3513/supp-1 Table S1 Figure 4 raw data Summary of the means of OPN expressions in percentages for Groups I, II and III at 6 weeks.

10.7717/peerj.3513/supp-2 Table S2 Figure 5 raw data Summary of the means of OC expressions in percentages for Groups I, II and III at 6 weeks.

10.7717/peerj.3513/supp-3 Table S3 Figure 7 raw data The percentage of OPN expression percentages in groups I, II and III at 12 weeks.

10.7717/peerj.3513/supp-4 Table S4 Figure 8 raw data The percentage of OC expressions percentages in groups I, II and III at 12 weeks.

## Additional Information and Declarations

### Data Availability

The following information was supplied regarding data availability: The raw data has been provided as Supplemental Files.
