# Supplementary material for: Local application of osteoprotegerin-chitosan gel in critical-sized defects in a rabbit model
Source: PeerJ. 2017 Jun 30;5:e3513. doi: 10.7717/peerj.3513 (PMC5494162; doi:10.7717/peerj.3513)
Supplement: Table S1 — Summary of the means of OPN expressions in percentages for Groups I, II and III at 6 weeks. [file peerj-05-3513-s001.docx]

**Raw Data**

Figures 4 summarized the means of OPN expressions in percentages for Groups I, II and III at 6 weeks.

| Groups | Measure 1 | Measure 2 | Measure 3 | mean | std |
| --- | --- | --- | --- | --- | --- |
| Group I | 41.3 | 38.5 | 43 | 40.93333 | 2.272297 |
| Group II | 52.2 | 56.1 | 62.2 | 56.83333 | 5.040172 |
| Group III | 72 | 70 | 76 | 72.66667 | 3.05505 |
