# Supplementary material for: Local application of osteoprotegerin-chitosan gel in critical-sized defects in a rabbit model
Source: PeerJ. 2017 Jun 30;5:e3513. doi: 10.7717/peerj.3513 (PMC5494162; doi:10.7717/peerj.3513)
Supplement: Table S2 — Summary of the means of OC expressions in percentages for Groups I, II and III at 6 weeks. [file peerj-05-3513-s002.docx]

**Raw Data**

Figure 5 raw data summarized the means of OC expressions in percentages for Groups I, II and III at 6 weeks.

| Groups | Measure 1 | Measure 2 | Measure 3 | mean | std |
| --- | --- | --- | --- | --- | --- |
| Group I | 45 | 44 | 47 | 45.33333333 | 2.272297 |
| Group II | 63 | 60 | 68 | 63.66666667 | 4.041451884 |
| Group III | 77 | 75 | 83 | 78.33333333 | 4.163332 |
