# Supplementary material for: Local application of osteoprotegerin-chitosan gel in critical-sized defects in a rabbit model
Source: PeerJ. 2017 Jun 30;5:e3513. doi: 10.7717/peerj.3513 (PMC5494162; doi:10.7717/peerj.3513)
Supplement: Table S3 — The percentage of OPN expression percentages in groups I, II and III at 12 weeks. [file peerj-05-3513-s003.docx]

**Raw Data**

Figure 7 raw data the percentage of OPN expression percentages in groups I, II and III at 12 weeks.

| Groups | Measure 1 | Measure 2 | Measure 3 | mean | std |
| --- | --- | --- | --- | --- | --- |
| Group I | 34.194 | 35.48 | 43.769 | 37.81433 | 5.196825 |
| Group II | 35.108 | 49.901 | 39.21 | 41.40633333 | 7.637154073 |
| Group III | 42.348 | 54.404 | 51.9 | 49.55066667 | 6.362099444 |
