# Supplementary material for: Local application of osteoprotegerin-chitosan gel in critical-sized defects in a rabbit model
Source: PeerJ. 2017 Jun 30;5:e3513. doi: 10.7717/peerj.3513 (PMC5494162; doi:10.7717/peerj.3513)
Supplement: Table S4 — The percentage of OC expressions percentages in groups I, II and III at 12 weeks. [file peerj-05-3513-s004.docx]

**Raw Data**

Figure 8 raw data The percentage of OC expressions percentages in groups I, II and III at 12 weeks.

.

| Groups | Measure 1 | Measure 2 | Measure 3 | mean | std |
| --- | --- | --- | --- | --- | --- |
| Group I | 18.275 | 35.4823.195 | 29.261 | 23.577 | 5.502953025 |
| Group II | 31.379 | 34.141 | 30.353 | 31.95766667 | 1.959177719 |
| Group III | 49.61 | 58.388 | 73.267 | 60.42166667 | 11.95889888 |
